# Outcomes and Prognostic Factors for Survival of Neonates With Necrotizing Enterocolitis

**DOI:** 10.3389/fped.2021.744504

**Published:** 2021-10-20

**Authors:** Elena Sophia Elekta Dilean Siahaan, Wahyudhy Adriansyah, Andika Purba Sasmita, Aditya Rifqi Fauzi, Andi Dwihantoro, Gunadi

**Affiliations:** Pediatric Surgery Division, Department of Surgery, Faculty of Medicine, Public Health and Nursing, Universitas Gadjah Mada/Dr. Sardjito Hospital, Yogyakarta, Indonesia

**Keywords:** developing country, necrotizing enterocolitis, prognostic factors, NEC staging, survival

## Abstract

**Background:** Due to the survival of preterm neonates being continually improved, the modifiable prognostic factors of necrotizing enterocolitis (NEC) are essential to be investigated and considered in making a suitable treatment to decrease the prevalence and effect of NEC. Many prognostic factors have been associated with the survival of neonates with NEC; however, the studies show conflicting results. Moreover, the study from developing countries regarding NEC outcomes is minimal. Here, we aimed to determine the survival of neonates with NEC and associate it with the prognostic factors.

**Methods:** A retrospective study was conducted using medical records of neonates with NEC at our institution from January 2014 to December 2019.

**Results:** Fifty-two neonates with NEC were involved with the overall survival of 44.2%. Log-rank analysis showed that NEC staging and birth weight were significantly associated with the survival of neonates with NEC with *a p*-value of 0.010 and 0.002, respectively, while sex, APGAR score, platelet count, and type of treatment were not (*p* = 0.068, 0.752, 0.087, and 0.343, respectively). Multivariate analysis revealed that sex and NEC staging were strongly associated with the survival of neonates with NEC with a *p*-value of 0.018 [HR = 3.10 (95% CI = 1.21–7.93)] and 0.019 [HR = 0.44 (0.22–0.87)], respectively.

**Conclusions:** Our study shows that sex and NEC staging might affect the survival of neonates with NEC. It implies that NEC staging should be closely monitored and intervened as early as necessary to prevent further morbidity and mortality.

## Introduction

Necrotizing enterocolitis (NEC) is the leading cause of morbidity and mortality of neonates with intestinal disorders in the neonatal intensive care unit (NICU) (1). Its incidence is 1 per 1,000 live births, and more than 90% of cases are premature infants (2). Its incidence is increasing because of the increasing number of preterm birth and the advances in neonatal care (3, 4).

Due to the continually improving survival of preterm neonates, the modifiable prognostic factors of NEC are essential to be investigated and considered in making a suitable treatment to decrease the prevalence and effect of NEC (4). Many prognostic factors have been associated with the survival of neonates with NEC; however, the studies show conflicting results (1, 5, 6). Moreover, the study from developing countries regarding NEC outcomes is minimal (7, 8). Here, we aimed to determine the survival of neonates with NEC and associate it with the prognostic factors.

## Methods

### Subjects and Necrotizing Enterocolitis Staging

A retrospective study was conducted using medical records of neonates with NEC at our institution from January 2014 to December 2019. We included 56 diagnosed with NEC, with the International Classification of Diagnosis (ICD) X code of P.77. According to modified Bell's staging, the diagnosis and staging of NEC were established, consisting of the severity of systemic, intestinal, radiographic, and laboratory findings (9). The exclusion criteria were incomplete medical records. We excluded four neonates due to incomplete medical records and investigated 52 neonates for final analysis.

The Ethical Committee of the Faculty of Medicine, Universitas Gadjah Mada/Dr. Sardjito Hospital, Indonesia, approved the study (KE/FK/0375/EC/2020).

### Prognostic Factors

We evaluated the following prognostic factors for the survival of neonates with NEC: sex, birth weight, NEC staging, platelet count, APGAR score, and type of treatment. Birth weight was classified into extremely low birth weight (<1,000 g), very low birth weight (<1,500 g), low birth weight (<2,500 g), and normal (≥2,500 g) according to World Health Organization classification, while the platelet count was defined as thrombocytosis (>350,000/mm^3^), normal (≥150,000–350,000/mm^3^), and thrombocytopenia (<150,000/mm^3^) according to a previous study (6). The type of treatment is divided into conservative and surgical procedures, while the APGAR Score was classified as asphyxia (<8) and non-asphyxia (≥8).

### Enteral Feeding

The decision to start enteral feeding was according to the following parameters: bowel sounds, no greenish gastric residual, and the volume of gastric residual was <1 ml/kg/day. Most of the enteral feeding was breastfeeding (94.2%) (Table 1).

**Table 1:** Baseline characteristics of neonates with NEC in our institution.

**Characteristics**	***N* (%)**
**Sex**	
Male	29 (55.8)
Female	23 (44.2)
**Asphyxia (APGAR score)**	
Yes (<8)	27 (51.9)
No (≥8)	25 (48.1)
**Birth weight (g)**	
Normal birth weight (≥2,500)	12 (23)
Low birth weight (<2,500)	20 (38.5)
Very low birth weight (<1,500)	17 (32.7)
Extremely low birth weight (<1,000)	3 (5.8)
**Platelet count (/mm** ^**3**^ **)**	
Thrombocytosis (>350,000)	6 (11.5)
Normal (≥150,000–350,000)	20 (38.5)
Thrombocytopenia (<150,000)	26 (50)
**NEC staging**	
IA	18 (34.6)
IB	2 (3.9)
IIA	15 (28.9)
IIB	0
IIIA	7 (13.4)
IIIB	10 (19.2)
**Type of treatment**	
Conservative	39 (75)
Operative	13 (25)
**Feeding**	
Breastfeeding	49 (94.2)
Formula	3 (5.8)
**Sepsis**	
Yes	48 (92.3)
No	4 (7.7)
**Mechanical ventilation**	
PCAC	4 (7.7)
PCMV	15 (28.8)
SIMV	2 (3.8)
NIMV	4 (7.7)
CPAP	10 (19.2)
**Survival**	
*Survived*	23 (44.2)
- Male	14 (60.9)
- Female	9 (39.1)
*Died*	29 (55.8)
- Male	14 (48.3)
- Female	15 (51.7)

*NEC, necrotizing enterocolitis*.

### Statistical Analysis

The survival of neonates with NEC was determined using a log-rank test, while the probabilities of the survival of the neonates were plotted using the Kaplan–Meier curve. The IBM SPSS Statistics version 16 (SPSS Chicago, IL, USA) was utilized to perform all statistical analyses.

## Results

### Baseline Characteristics

We involved 52 neonates with NEC with overall survival of 44.2%. Most of them were male (55.8%), with asphyxia (51.9%), low birth weight or less (77%), thrombocytopenia (50%), sepsis (92.3%), and breastfeeding (94.2%) (Table 1).

### Association Between Prognostic Factors and Survival of Neonates With Necrotizing Enterocolitis

**Figure 1:**
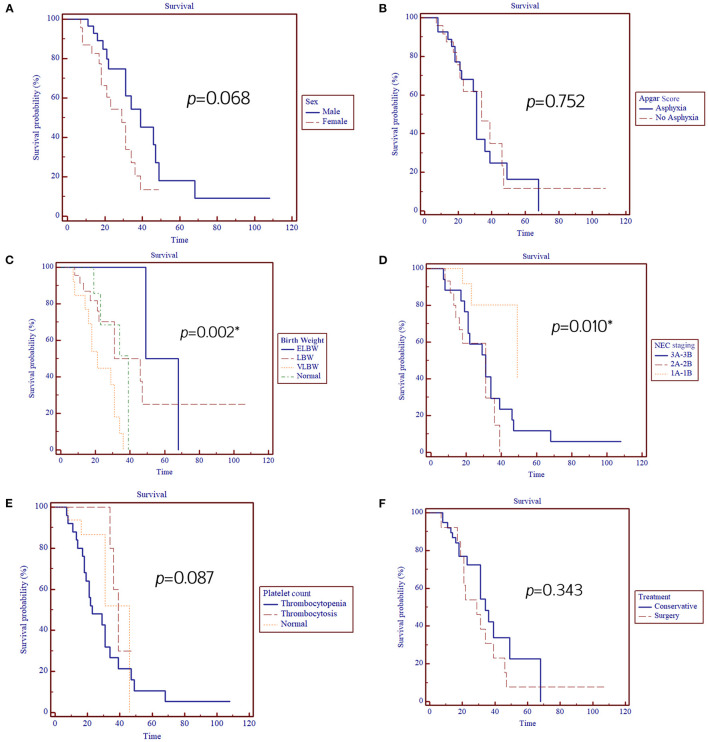
Kaplan–Meier analysis for the association between prognostic factors: **(A)** Sex, **(B)** APGAR score, **(C)** Birth weight, **(D)** NEC staging, **(E)** Platelet count, **(F)** Treatment, and NEC patients' survival. ^*^, significant *p* < 0.05.

Log-rank analysis showed that NEC staging and birth weight were significantly associated with the survival of neonates with NEC with *a p*-value of 0.010 and 0.002, respectively. At the same time, sex, APGAR score, platelet count, and type of treatment were not (*p* = 0.068, 0.752, 0.087, and 0.343, respectively) (Figure 1; Table 2).

**Table 2:** Association between prognostic factors and survival of neonates with NEC in our institution.

**Variables**	**HR (95% CI)**	***p*-Value**
**Sex**		
Female	1.89 (0.89–4.04)	0.068
**Asphyxia**		
APGAR score <8	1.12 (0.54–2.34)	0.752
**Birth weight (Ref: normal birth weight)**		
Low birth weight	0.87 (0.33–2.32)	0.002^*^
Very low birth weight	2.75 (0.83–9.13)	
Extremely low birth weight	0.45 (0.14–1.46)	
**Platelet count (Ref: Normal)**		
Thrombocytopenia	1.84 (0.75–4.53)	0.087
Thrombocytosis	0.62 (0.21–1.80)	
**NEC staging (Ref: IA-IB)**		
IIA–IIB	5.83 (2.07–16.40)	0.010^*^
IIIA–IIIB	3.89 (1.71–8.84)	
**Type of treatment**		
Surgery	1.41 (0.65–3.05)	0.343

* *p < 0.05; CI, confidence interval; HR, hazard ratio; NEC, necrotizing enterocolitis; Ref, reference*.

### Multivariate Analysis of Prognostic Factors for Survival of Neonates With Necrotizing Enterocolitis

Multivariate analysis revealed that sex (female) and NEC staging were strongly associated with the survival of neonates with NEC with *a p*-value of 0.018 {hazard ratio [HR] = 3.10 [95% confidence interval (CI) = 1.21–7.93]} and 0.019 [HR = 0.44 [95% CI = 0.22–0.87)], respectively (Table 3).

**Table 3:** Multivariate analysis of survival of neonates with NEC in our institution.

**Variables**	**HR (95% CI)**	***p*-value**
Sex (female)	3.10 (1.21–7.93)	0.018^*^
Asphyxia	1.04 (0.36–2.98)	0.940
Birth weight	1.41 (0.82–2.41)	0.211
Platelet count	1.94 (0.93– 4.06)	0.079
NEC staging	0.44 (0.22–0.87)	0.019^*^
Type of treatment	1.84 (0.54–6.34)	0.332

* *p < 0.05; CI, confidence interval; HR, hazard ratio; NEC, necrotizing enterocolitis*.

## Discussion

Here, we show that the overall survival of our NEC neonates is 44.2%, which is similar to a previous study (10). One reason for the high mortality rate was withdrawal from critical care, while another reason was the unavailability of pediatric surgery services in some hospitals; thus, surgery was not possible (10). Our institution, as an academic referral hospital, has pediatric surgery services. However, our findings were not compatible with a previous report (10) since the type of treatment did not affect the outcome of NEC infants (Table 3).

We reveal that NEC staging is a strong prognostic factor for the survival of neonates with NEC. Our findings were compatible with a previous study that revealed that the NEC stage III in VLBW and LBW infants is an independent prognostic factor for the survival of neonates with NEC (7). Moreover, lower birth weight was significantly associated with NEC incidence and mortality (11, 12). Immaturity of the gastrointestinal tract, digestive function, circulation regulation, barrier function, and immune defense are essential factors in explaining NEC occurring in infants with LBW (13). We show new evidence to support this hypothesis by providing data from a population ethnically different from the previous report (7). The previous report showed that the prognostic factors for NEC had been expanded, including ethnicity (4). Moreover, due to the survival of preterm neonates being continually improved, it is suggested that clinicians and researchers should look for the prognostic factors for NEC, particularly the modifiable one, to be taken into consideration in making a suitable treatment to decrease the prevalence and effect of NEC (4).

Interestingly, female patients had a 3.1-fold higher risk of mortality than male patients. A previous study showed that male is a risk factor for mortality (11). These differences might be due to different ethnicities. Ethnicity has been considered as a prognostic factor for NEC (4). In addition, a previous study showed no association between sex and NEC (9). They suggested continuous assessment on the impact of sex on the severity of NEC since the male has tended to suffer from NEC (9).

Our study presented that thrombocytopenia almost reached a significant level affecting the mortality of NEC infants with the HR of ~2 (*p* = 0.07) (Table 3). Most neonates with advanced stages of NEC will have thrombocytopenia within 24–72 h of disease onset (14). Thrombocytopenia level is strongly correlated with the clinical staging of NEC, and a progressive decrease in thrombocyte level implies the development of intestinal gangrene (15). In addition, thrombocytopenia has been shown as a strong predictor of the mortality of neonates with NEC (16).

Treatment type is a significant prognostic factor in NEC patients (17). Infants with NEC who underwent surgery had higher morbidity and mortality than those who received conservative treatment (17, 18). However, our findings showed that type of treatment did not affect the mortality of NEC neonates. While Hull et al. (19) revealed that different surgical approaches affected the mortality of NEC neonates, none of the specific surgical approaches is suggested for NEC (19). It depends on several variables, including the birth weight, hemodynamic status, comorbidities, existing resources, intraoperative findings, and attending physician preference of the neonate (19).

Our study noted several limitations, including a small sample size and a single-center report, implying that a further multicenter study with larger sample size is necessary to clarify and confirm our findings. These weaknesses should be noted during the interpretation of our findings. Due to its retrospective design, we have difficulty evaluating the long-term complications of NEC, including neurodevelopmental impairment, poor growth, gastrointestinal sequels, such as strictures, adhesions, feeding difficulties, cholestasis, short bowel syndrome, and intestinal failure (20).

## Conclusion

Our study shows that sex and NEC staging might affect the survival of neonates with NEC. It implies that NEC staging should be closely monitored and intervened as early as necessary to prevent further morbidity and mortality.

## Data Availability Statement

The original contributions presented in the study are included in the article/supplementary material, further inquiries can be directed to the corresponding author/s.

## Ethics Statement

The studies involving human participants were reviewed and approved by Faculty of Medicine, Public Health and Nursing, Unversitas Gadjah Mada/Dr. Sardjito Hospital. Written informed consent to participate in this study was provided by the participants' legal guardian/next of kin.

## Author Contributions

AD and G conceived the study. ESEDS, WA, APS, ARF, and G drafted the manuscript. ESEDS and G analyzed the data. AD and G facilitated all project-related tasks. All authors read and approved the final manuscript.

## Conflict of Interest

The authors declare that the research was conducted in the absence of any commercial or financial relationships that could be construed as a potential conflict of interest.

## Publisher's Note

All claims expressed in this article are solely those of the authors and do not necessarily represent those of their affiliated organizations, or those of the publisher, the editors and the reviewers. Any product that may be evaluated in this article, or claim that may be made by its manufacturer, is not guaranteed or endorsed by the publisher.

## Abbreviations

APGAR: appearance pulse grimace activity respiration
CI: confidence interval
HR: hazard ratio
NEC: necrotizing enterocolitis
NICU: neonatal intensive care unit.
